# LGBTQ+ related social media and website communication before and after 2025 executive orders from the top 50 ranked US medical schools

**DOI:** 10.3389/fsoc.2026.1875398

**Published:** 2026-09-03

**Authors:** Sophia D. Bodor, Nicholas Musinguzi, Benjamin W. Langworthy, Michelle N. Rheault

**Affiliations:** 1 Wellesley College, Wellesley, MA, United States; 2 Division of Biostatistics and Health Data Science, School of Public Health, University of Minnesota, Minneapolis, MN, United States; 3 Department of Pediatrics, University of Minnesota Medical School, Minneapolis, MN, United States; 4 Center for Women in Medicine and Science, University of Minnesota Medical School, Minneapolis, MN, United States

**Keywords:** academic medicine, diversity, equity, inclusion, LGBTQ+, social media

## Abstract

**Purpose:**

We assessed changes in US medical schools’ social media communication regarding LGBTQ+ topics and changes in website Diversity, Equity, and Inclusion (DEI) related content after the January 2025 anti-DEI executive orders.

**Method:**

Social media profiles from the top fifty medical schools as defined by the 2024 Blue Ridge Institute for Medical Research were reviewed from Feb–June 2024 and Feb–June 2025 and the number of posts featuring LGBTQ+ content was compared. Platforms included X and Instagram. Medical school websites were reviewed during pre- and post-executive orders timeframes and the presence of a DEI office or diversity statement was compared. We evaluated by linear regression whether results correlated with the medical school state’s 2024 presidential voting record.

**Results:**

The average number of LGBTQ+ related posts per medical school decreased from 3.10 to 0.88 (*p* < 0.001) from 2024 to 2025. The presence of a DEI office decreased from 94 to 46% from 2024 to 2025 (*p* < 0.001). Similarly, 94% of medical schools had a diversity statement posted on their website in 2024 compared to 50% in 2025 (*p* < 0.001). Medical schools located in states that voted for the Republican candidate in 2024 had fewer LGBTQ+ related posts in both time periods.

**Conclusion:**

We found a significant decrease in medical school LGBTQ+ social media content, publicized DEI offices, and published diversity statements after the anti-DEI executive orders of 2025. These changes may impact medical school climate for LGBTQ+ students and faculty and the impact of these changes requires further study.

## Introduction

Over the past decade, medical schools have been increasing their efforts to create diverse communities of students, graduate medical trainees, and faculty for a number of reasons. Healthcare organizations with a highly diverse workforce have better patient care outcomes, improvements in health equity, and higher impact research results (Marrast et al., 2014; Tsugawa et al., 2017; AlShebli et al., 2018). Sexual orientation and gender identity are important, but often invisible, components of diversity among medical school faculty. Lesbian, gay, bisexual, transgender, and queer (LGBTQ+) individuals remain underrepresented among US medical school faculty, accounting for only 3.9% according to a 2022 Association of American Medical Colleges (AAMC) report (AAMC, 2022). In non-medical fields, organizations with diversity of sexual orientation among employees and proactive strategies to strengthen diversity consistently outperform those that are not as diverse (Cunningham, 2011). Organizations that openly communicate their support of LGBTQ+ employees using an “identity-conscious” approach attract more LGBTQ+ employees and have better retention (Mor et al., 2025). A diverse environment is vital for medical student well-being as well. Approximately 30% of sexual and gender minority medical students conceal their sexual identify during medical school with many students identifying lack of support and local social and cultural norms as part of the reason (Mansh et al., 2015).

Public social media profiles and official websites are two components that medical schools can utilize to highlight the importance of diversity for prospective students, trainees, or faculty in order to demonstrate an inclusive climate and increase recruitment and retention (Campbell et al., 2023; Naaseh et al., 2021). Social media posts can improve engagement and can show a school’s commitment to diversity, equity, and inclusion (DEI) (Bhalla, 2019). Contemporary LGBTQ+ communities have a unique relationship with social media that centers on community building and a sense of belonging (Eickers, 2024). When medical schools include LGBTQ+ topics and highlight LGBTQ+ faculty on their social media platforms, they are signaling similar values of inclusion and belonging. Posting a school’s diversity statement and DEI office information on their website is another publicly facing affirmation of institutional support for underrepresented groups, although these have limitations (Carnes et al., 2019).

Over the past decade, a majority of medical schools developed institutional diversity statements and DEI offices to highlight their commitment to DEI concepts (Stowers et al., 2026; Carnes et al., 2019). With a change in the United States federal administration in early 2025, there was an effort to eliminate DEI programs via multiple executive orders (Trump, 2025b, 2025c). LGBTQ+ individuals were specifically targeted in multiple additional executive orders aiming to eliminate funding for gender-affirming care and to reevaluate Title VII protections based on gender identity (Trump, 2025a, 2025d). In response to these executive orders, the National Institutes of Health Sexual & Gender Minority Research Office was dismantled and medical schools saw research grants related to LGBTQ+ health and other health equity topics canceled or put on hold with threats of further funding cuts or penalties if DEI programs were maintained (Reardon, 2025).

Given the role of social media postings and formal DEI programs in maintaining an inclusive culture at medical schools, we performed this study to assess whether there were changes in medical schools’ social media communication regarding LGBTQ+ topics or changes in website DEI related content after the anti-DEI and anti-LGBTQ+ executive orders. In addition, we aimed to determine if these factors were associated with the medical school’s state 2024 presidential election results as a surrogate for their local political climate.

## Methods

This was a cross-sectional observational study of medical school social media accounts and websites before and after executive administration changes in January 2025. The top fifty medical schools as defined by the Blue Ridge Institute for Medical Research, 2024 Rankings of NIH Funding were identified (Blue Ridge Institute for Medical Research, 2024; !Supplemental Table S1). The Blue Ridge ranked schools were chosen to define an objective subset of US medical schools for analysis that were likely to be impacted by federal regulations given their large research portfolios. Each school’s official social media accounts (main medical school account and DEI office account if present) including X (formerly Twitter) and Instagram were reviewed to identify posts featuring LGBTQ+ content as the main focus of the post. Relevant reposts from other accounts were included in this count. We define “LGBTQ+ content” to be a post that shares information about an LGBTQ+ event, holiday, health initiative/study, or content that showcases profiles of identified LGBTQ+ patients or faculty members. Social media posts were reviewed from two time periods: 2/1/24–6/30/24 and 2/1/25–6/30/25. Social media profiles were accessed between 6/23/25–8/5/25. Posts featuring LGBTQ+ content were subdivided into two categories: posts directed toward patients and others. “Posts directed toward patients” was defined as posts to increase patient outreach or involvement with the medical school including clinical trial enrollment, clinic offerings, health advice, and patient testimonies. “Posts directed toward others” was defined as all other posts targeting prospective faculty and students that were not directly related to patient care. All accounts were reviewed by a single individual (SB) and adjudicated by the senior author if necessary (MR).

Public websites from the above fifty medical schools were reviewed to identify the presence of an office of DEI or similar and a diversity statement. Similar to a mission statement, a diversity statement represents an institution’s long-term commitment to improving diversity, equity, and inclusion for its employees and students. A diversity statement is different from Title IX and other legally binding non-discrimination statements because it reflects a voluntary and comprehensive strategy for diversity and inclusion that goes beyond federal guidelines.

We did not include legal Title IX statements or nondiscrimination statements as “diversity statements” in this study. Websites were reviewed at two different time points to capture the findings before and after the January 2025 executive orders referenced above. Websites were accessed between the dates of 7/7/25–8/5/25 to reflect the post-executive order timeframe. To review websites from before 2025, the Wayback Machine, an open-source service from the Internet Archive, was used to view the page (Internet Archive, 2025; Asthana et al., 2025). We selected the page capture dated closest to 6/30/24 as the baseline.

Each medical school was classified based on which party’s candidate won the popular vote in the 2024 presidential election as either a red state (Republican) or blue state (Democratic).

The number of social media posts containing LGBTQ+ content was summarized using the mean and standard deviation. We tested for the difference in average number of posts between 2/1/24–6/30/24 and 2/1/25–6/30/25 using a paired t-test. This analysis was repeated for the main medical school account and the DEI specific account. In addition, we fit two generalized estimating equation (GEE) models using an identity link with the number of posts across the main accounts and DEI specific accounts as the outcome, and each school treated as a cluster. We used an exchangeable working correlation matrix and report the robust sandwich variance estimates. We also considered a sensitivity analysis using a Poisson GEE model with a log link function. In the first model the covariates were year (2024 vs. 2025) and a binary indicator of whether Trump won the state the school was located in for the 2024 presidential election. For the second model the covariates were year (2024 vs. 2025) and the percentage of the two-way vote received by Trump in the state the school was located in. The percentage of the two-way vote only included those who voted for Trump or Harris and excluded any third party or other votes.

We reported the percentage of schools with a DEI office and diversity statement in 2024 and 2025 and used a McNemar test to test for differences between 2024 and 2025. The McNemar test was used because it is a non-parametric test that can account for the paired nature of the data, where each school’s data for 2024 and 2025 represents a paired set of observations. We then fit (GEE) models using a logistic link function with the presence of a DEI office as the outcome, and each school treated as a cluster. These models also an exchangeable working correlation matrix and the robust sandwich variance estimator. Similar to the linear models with number of posts as the outcome, in the first model the covariates were year (2024 vs. 2025) and a binary indicator of whether Trump won the state the school is located in for the 2024 presidential election. In the second model the covariates were year (2024 vs. 2025) and the % of the two-way vote received by Trump in the state the school is located in.

## Results

### Social media

There were 104 X and Instagram accounts representing 49 medical schools and their representative offices of DEI that were analyzed for posts containing LGBTQ+ content between February and June 2024 and 2025. One school was excluded because of their lack of X or Instagram accounts.

The number of LGBTQ+ relevant social media posts per medical school varied widely in each time period from 0 to 12. The mean number of LGBTQ+ posts per medical school in the 2024 period was 3.10 (SD 3.53). There was a significant reduction in LGBTQ+ posts in the 2025 time period to a mean of 0.88 (SD 1.86, *p* < 0.001; Table 1). These results were similar when evaluating main university medical school accounts or DEI specific accounts (Table 1). LGBTQ+ posts targeted specifically to patients were rarely observed in either time period. In 2025, only 31% of medical schools or DEI offices posted any LGBTQ+ content compared to 71% in 2024.

**Table 1:** The average number of LGBTQ+ social media posts from February to June 2024 versus 2025.

Location of social media posts	Number of posts (mean, SD) Feb–June 2024	Number of posts (mean, SD) Feb–June 2025	*p* value
Main + DEI accounts	3.10 (3.53)	0.88 (1.86)	<0.001
Main medical school accounts	2.51 (3.04)	0.71 (1.73)	<0.001
Medical school DEI accounts	0.59 (1.86)	0.16 (0.72)	0.04
Patient targeted posts for main + DEI accounts	0.18 (0.39)	0.06 (0.24)	0.03

Twenty-one (42%) of medical schools reviewed were located in states that voted for Donald Trump in the 2024 presidential election (red states) and 58% were located in states that voted for Kamala Harris (blue states). We next evaluated by linear regression whether medical school LGBTQ+ content social media posts correlated with the state voting record. We found that medical schools in red states had an average of 1.27 fewer LGBTQ+ content posts compared to blue states adjusting for year (CI = [−2.48, −0.07], *p* = 0.039). Each additional 10% in Trump vote share was associated with a reduction of 0.8 in the number of LGBTQ+ posts regardless of year (CI = [−1.60, −0.1], *p* = 0.026). Results from the Poisson GEE model showed similar results in the direction of effect, however the effect for red states vs. blue states was no longer statistically significant. The effect for the percentage of Trump vote was still statistically significant.

### Website communications

**Figure 1:**
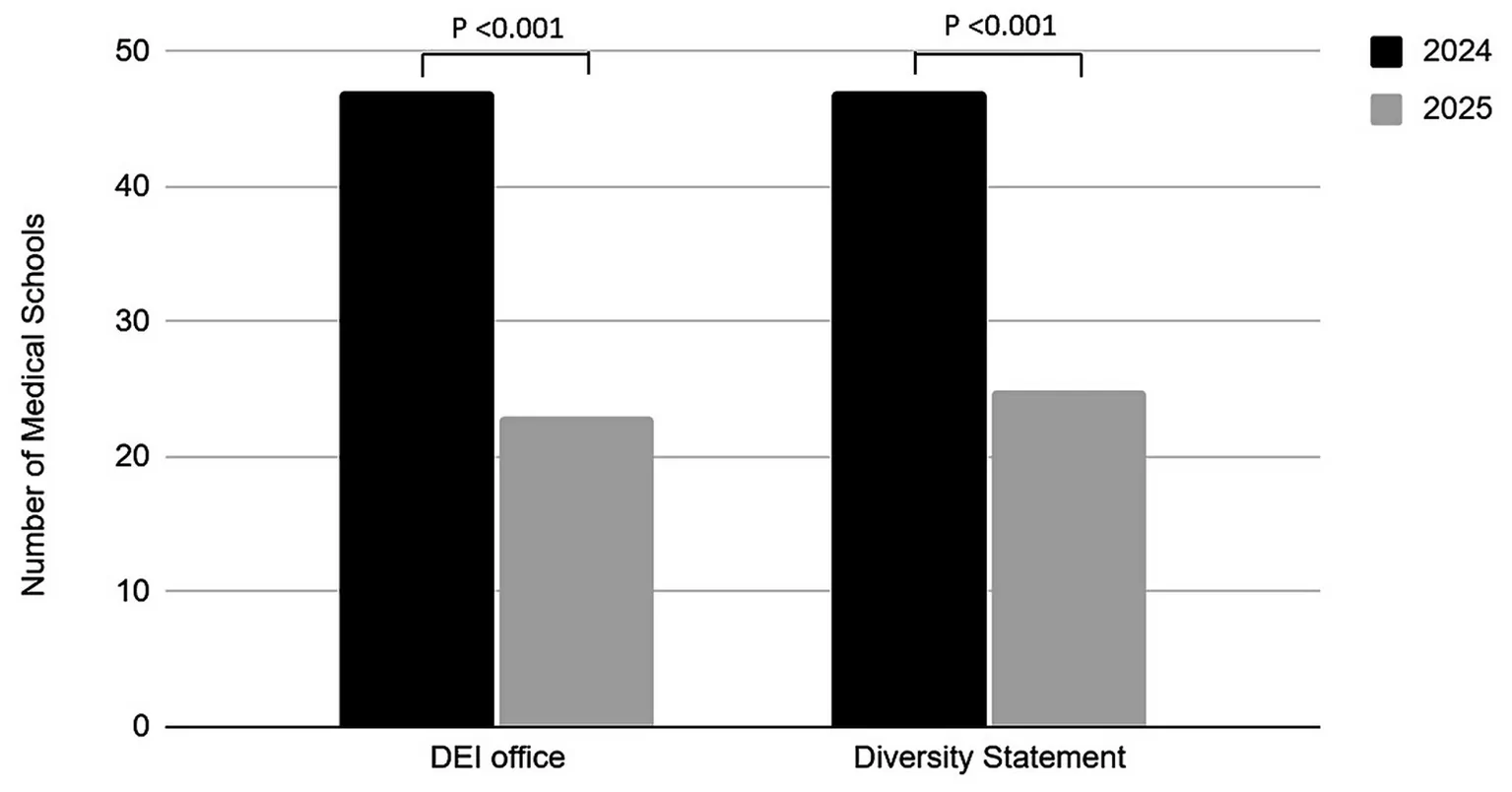
Medical school website inclusion of DEI Office and diversity statements comparing 2024 and 2025.

The websites of 50 medical schools were analyzed both before and after the January 2025 executive orders. We found that 94% of medical schools included a DEI office on their websites in 2024 compared to 46% in 2025 (*p* < 0.001; Figure 1). Similarly, 94% of medical schools had a diversity statement posted on their website in 2024 compared to 50% in 2025 (*p* < 0.001). Only 28% of the diversity statements in 2024 included explicit protections for sexual orientation and gender identity.

Logistic regression was performed comparing the presence of a DEI office on medical school websites by state voting record as well as by the percentage of Trump votes by state in 2024. The odds of having a DEI office were lower among red states controlling for year (OR 0.23, 95% CI 0.07, 0.72; *p* = 0.012). The odds of having a DEI office in 2024 was significantly higher than 2025, even after controlling for state voting record (OR 24.09, 95% CI 7.70, 75.36; *p* < 0.001). Each additional percentage of Trump vote was associated with a reduction in the odds of having a DEI office on the website after controlling for the year (*p* = 0.012).

## Discussion

We hypothesized that social media posts with LGBTQ+ content at medical schools would significantly decrease after the anti-DEI executive orders in January 2025 and this was confirmed by our analysis. We were unable to identify studies that examined changes in other DEI components such as race or ethnicity for comparison. Social media accounts are highly public forms of outreach from medical schools to their students, faculty, and the community they serve. Based on our results, there was a statistically significant decrease in posts among all medical schools, even when comparing posts by schools in red states and in blue states.

The overall decline in number of DEI-based social media accounts and fewer LGBTQ+ inclusive posts may negatively impact how people view a medical school’s LGBTQ+ inclusivity. The dismantling of social media accounts tied to diversity offices or initiatives and the general trend of decreased posts highlighting the LGBTQ+ community may have negative impacts on feelings of LGBTQ+ inclusion at medical schools. Public signals of LGBTQ+ support and acceptance can affect the openness of LGBTQ+ community members about their identity in the workplace. A possible decrease in LGBTQ+ enrollment or “out” LGBTQ students and faculty in medical schools can have a negative effect on the mental health, belonging, and work satisfaction of those students or faculty and on the culture of inclusivity of the medical school (Cook et al., 2025; Pagliaccio, 2025).

Our second hypothesis was that the number of publicly available diversity statements and DEI offices at each medical school would decrease significantly given concerns about loss of research funding from the federal government. We observed a reduction in public DEI office websites from 94% in 2024 to 45% in 2025. A recent study of 195 US medical schools identified a similar number of medical school websites with DEI office information in July 2025 (40%), however no comparison was made in that study to the time period before the January 2025 anti-DEI executive orders (Sreepada and Ko, 2026).

Diversity mission statements are common among US public and private medical schools (Stowers et al., 2026). We observed a reduction in public medical school diversity statements from 94% in 2024 to 50% in 2025.

Medical schools in red states, specifically those with higher percentages of Trump voters, were least likely to have a DEI office after the executive orders. The second Trump administration’s anti-diversity policies are not the start of this trend. Rather, they are part of a broader movement to eliminate diversity initiatives in education that started in 2022 with Florida’s passage of the Parental Rights in Education Act (Florida House Bill 1557) commonly known as the “Don’t Say Gay” law.

There are several limitations to this work. This study looks at the public, outward communications made by medical schools which does not account for the internal perceptions of DEI at these schools. This study cannot account for deleted posts or social media accounts, which may affect the post counts in both time spans. In addition, the total number of social media posts per school in each time period was not determined, thus decreases in LGBTQ+ posts may have been due to an overall reduction in posts and not content specific. An additional limitation is that social media posts were adjudicated by a single individual, although clear guidelines were used to identify LGBTQ+ posts for inclusion. We acknowledge that a diversity office or diversity statement may have been present, but simply removed from the website due to concerns about visibility in the current political climate. We believe that the lack of a public facing DEI office, even if one still exists, sends a strong message to potential students and faculty about medical school priorities. An additional limitation is that website content may have been missed due to use of the Wayback Machine to identify past websites. The Wayback Machine collects manual captures of webpages, therefore the number of captures and span of time collected for each page can vary widely. Finally, the medical schools included in the list of the top fifty schools by NIH Blue Ridge ranking may not be representative of all US medical schools. These schools may be enriched for those more likely to change LGBTQ+ content social media posts or website inclusion of a DEI office or diversity statement to align with federal agency regulations and maintain research funding.

In summary, we found a significant decrease in LGBTQ+ content on medical school social media accounts after the anti-DEI executive orders of 2025 that correlated with a state’s voting record. In addition, we found a significant reduction in the percentage of medical schools that had diversity statements and DEI offices on their websites. These changes may impact medical school climate for LGBTQ+ students and faculty and the impact of these changes requires further study.

## Data Availability

The raw data supporting the conclusions of this article will be made available by the authors, without undue reservation.
