# Supplementary material for: LGBTQ+ related social media and website communication before and after 2025 executive orders from the top 50 ranked US medical schools
Source: Front Sociol. 2026 Sep 3;11:1875398. doi: 10.3389/fsoc.2026.1875398 (PMC13581799; doi:10.3389/fsoc.2026.1875398)
Supplement: Supplementary file 1 [file Table_1.DOCX]

Supplemental Table S1: Blue Ridge Institute for Medical Research Rankings of Top 50 Medical Schools for NIH Funding in 2024

University of California, San Francisco

Washington University in St. Louis

Yale University

University of Pennsylvania

Vanderbilt University

Johns Hopkins University

Stanford University

University of Pittsburgh

Columbia University

University of Michigan

New York University

Mount Sinai School of Medicine

Duke University

University of California, San Diego

Northwestern University

University of California, Los Angeles

University of Washington

Emory University

University of North Carolina

Baylor College of Medicine

Cornell University

University of Colorado, Denver

University of Wisconsin, Madison

University of Texas Southwestern

University of Minnesota

Case Western Reserve University

Mayo Clinic

University of Alabama

University of Chicago

Indiana University

Oregon Health and Sciences

University of Southern California

University of California, Davis

University of Utah

Albert Einstein College of Medicine

University of Massachusetts Worcester

Ohio State University

University of California, Irvine

University of Virginia

University of Maryland

University of Florida

University of Miami

Harvard Medical School

University of Rochester

Boston University

University of Iowa

Rutgers University

Wake Forest University

University of Texas, San Antonio

University of Illinois Chicago
